# Flow cytometric probing of mitochondrial function in equine peripheral blood mononuclear cells

**DOI:** 10.1186/1746-6148-3-25

**Published:** 2007-09-28

**Authors:** Dominique Cassart, Thomas Fett, Michaël Sarlet, Etienne Baise, Freddy Coignoul, Daniel Desmecht

**Affiliations:** 1 Department of Pathology, Faculty of Veterinary Medicine, University of Liege, Sart Tilman B43, B-4000 Liège, Belgium

## Abstract

**Background:**

The morphopathological picture of a subset of equine myopathies is compatible with a primary mitochondrial disease, but functional confirmation in vivo is still pending. The cationic dye JC-1 exhibits potential-dependent accumulation in mitochondria that is detectable by a fluorescence shift from green to orange. As a consequence, mitochondrial membrane potential can be optically measured by the orange/green fluorescence intensity ratio. A flow cytometric standardized analytic procedure of the mitochondrial function of equine peripheral blood mononuclear cells is proposed along with a critical appraisal of the crucial questions of technical aspects, reproducibility, effect of time elapsed between blood sampling and laboratory processing and reference values.

**Results:**

The JC-1-associated fluorescence orange and green values and their ratio were proved to be stable over time, independent of age and sex and hypersensitive to intoxication with a mitochondrial potential dissipator. Unless time elapsed between blood sampling and laboratory processing does not exceed 5 hours, the values retrieved remain stable. Reference values for clinically normal horses are given.

**Conclusion:**

Whenever a quantitative measurement of mitochondrial function in a horse is desired, blood samples should be taken in sodium citrate tubes and kept at room temperature for a maximum of 5 hours before the laboratory procedure detailed here is started. The hope is that this new test may help in confirming, studying and preventing equine myopathies that are currently imputed to mitochondrial dysfunction.

## Background

Equine atypical myopathy affects horses and ponies kept on pasture and not exercised either prior to or at the time of the first clinical signs. All cases have been reported in the springtime or autumn and after a sudden drop in the minimum daily temperature [1,2]. Affected horses are usually well and suddenly display signs of acute myopathy (stiffness, muscle pain, muscle fasciculations, abnormal gait, recumbency, myoglobinuria, tachycardia, sweating), with no hyperthermia and unchanged appetite [3]. Unlike the other types of myopathy, this condition is often fatal within 12 to 72 hours. Blood analysis consistently shows a spectacular increase in muscle enzymes, in particular the creatine-kinases [3]. Recently, a detailed macroscopical, histopathological, enzymohistochemical and ultrastructural study of 32 clinical cases was reported [2]. The morphopathological picture of the disease suggested a mitochondrial disorder the characteristics of which are compatible with those of equine toxic myopathies due to plant, bacterial or fungal toxins [4-9]. Although morphologic clues of mitochondrial dysfunction in these equine diseases have accumulated, a functional confirmation is still pending. Also, as the clinical diagnosis is only possible late in the course of these diseases, when definitive lesions have occurred, an early marker is crucially needed to improve the clinical prognosis of established cases and to detect subclinical cases that would benefit of preventive measures. Epidemiological studies aimed at delineating the precise environmental conditions that increase the risk of developing atypical myopathy would also gain from a standardized laboratory test that lends itself to mass screening. Therefore, reliable methods for the sensitive determination of a possible alteration of mitochondrial function in equine tissues are desirable.

The cationic dye JC-1 exhibits potential-dependent accumulation in mitochondria, indicated by a fluorescence shift from green (~529 nm) to orange (~590 nm). This potential-sensitive color shift is due to the concentration-dependent intramitochondrial formation of orange fluorescent oligomers [10-12]. As a consequence, mitochondrial membrane potential (Δψ_m_) can be optically measured by the orange/green fluorescence intensity ratio. This ratio is dependent only on the Δψ_m _and not on other factors such as mitochondrial size, shape, and density, which renders JC-1 far more reliable than any other single-component fluorescence signals to evaluate mitochondrial function. JC-1 orange-green fluorescence ratio has been used as an indicator of mitochondrial potential in isolated mitochondria [13] but also in intact tissues [14], spermatozoa [15] and cell lines, including myocytes [12] and neurons [16]. Very subtle heterogeneity in cellular responses were already discerned in this way [11,13,16], the most widely implemented application being for detection of mitochondrial depolarization occurring during apoptosis [17-20]. This series of studies prompted us to suggest that JC-1-associated orange/green ratio could function as a marker of mitochondrial dysfunction in the clinic. Since mitochondrial dysfunction precedes morphologic alterations, it would permit an early diagnosis of mitochondria-associated rhabdomyolyses in horses. Although muscle biopsies would directly target the tissue of choice, questions remains about the muscle to be sampled [2]. Furthermore, multiple biopsies should be avoided in the routine clinic due to animal welfare concerns. These practical considerations led us to evaluate the candidature of JC-1 as an indicator of mitochondrial potential in equine peripheral blood mononuclear cells. In this paper, a standardized analytic procedure is proposed along with a critical appraisal of the crucial questions of reproducibility, effect of time elapsed between blood sampling and laboratory processing and reference values.

## Results

**Figure 1:**
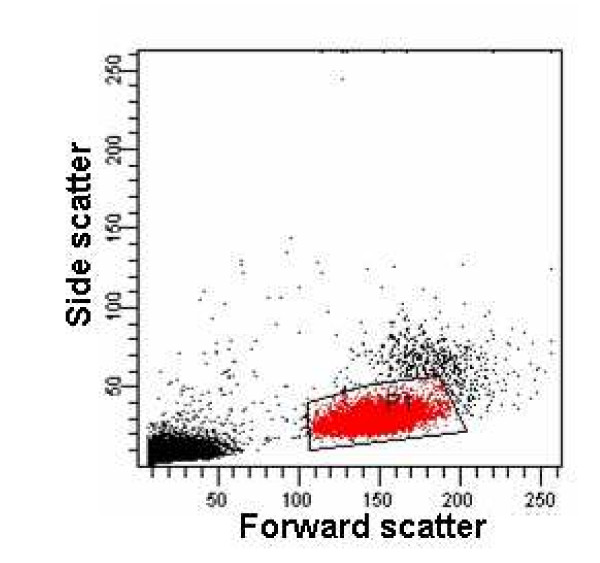
Forward vs. side scatter plot of equine PBMC suspension in PBS. The cell subpopulation typically analysed for JC-1-associated fluorescences is marked by the polygon area. Although more dispersed, a second subpopulation is readily visible.

Flow cytometric analysis of physical characteristics of equine PBMC, using forward (FSC) and side (SSC) scatters, consistently revealed two distinct subpopulations (Fig. 1). The first is characterized by low FSC and SSC and presumably corresponds to lymphocytes. Subsequent analysis of fluorescent labelling of PBMC using appropriate monoclonal antibodies confirmed that this population indeed consisted of lymphocytes. The second subpopulation, showing slightly higher FSC and SSC, is rather compatible with monocytes. Because the first subpopulation was always less scattered than the second, it was chosen to gate the cell subpopulation of which the JC-1-associated fluorescences were studied.

**Figure 2:**
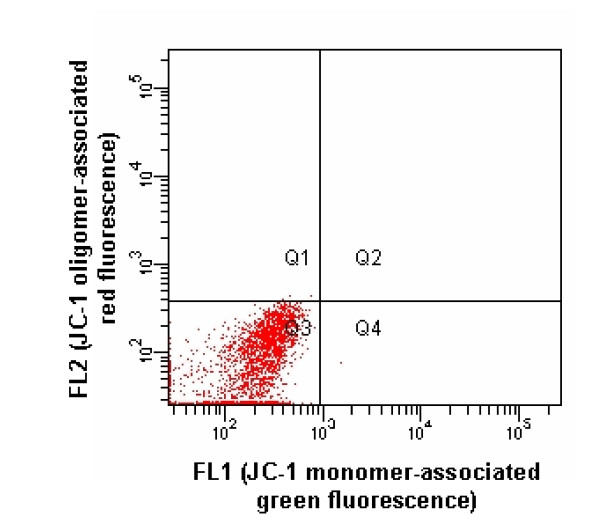
FL2 vs. FL1 scatter plot showing autofluorescence emission of cell subpopulation gated in Fig. 1 under 488 nm Sapphire™ solid-state laser excitation.

**Figure 3:**
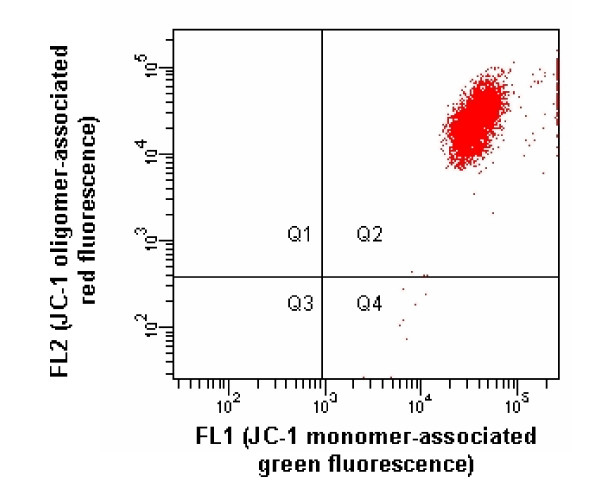
FL2 vs. FL1 scatter plot showing JC-1-associated fluorescence emission of cell subpopulation gated like in Fig. 1 under 488 nm Sapphire™ solid-state laser excitation.

**Figure 4:**
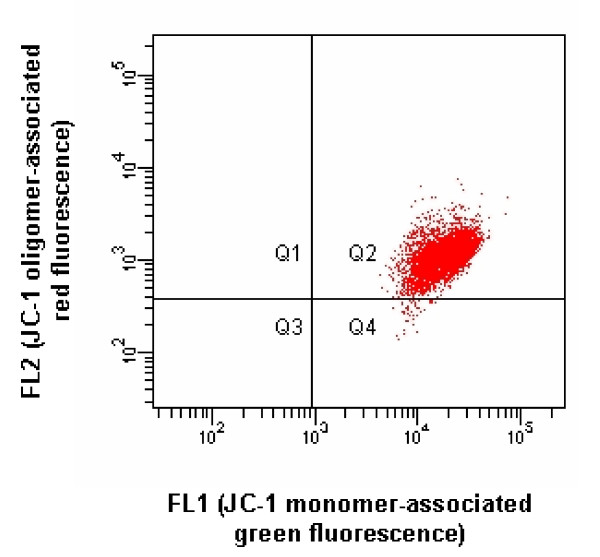
FL2 vs. FL1 scatter plot showing JC-1-associated fluorescence emission of cell subpopulation gated like in Fig. 1, after intoxication with the Δψ_m _dissipator CCCP (100 μM).

Using 488 nm Sapphire™ solid-state laser excitation, the gated nonstained cell populations showed readily identifiable and very homogeneous autofluorescent emissions within the 530/30 and 585/42 nm bandpass windows (Fig. 2). However, the gated stained cell populations displayed much more intense fluorescences in both windows (Fig. 3), which yielded an excellent signal-to-noise ratio. Among the ~120 stained cell populations of which the results contributed to this study, there was never > 1% of cells falling in the green autofluorescence window, showing that the JC-1 concentration used is sufficient to stain all cells despite the unavoidable slight variation of the number of cells between cell suspensions. For the orange autofluorescence, identical results were obtained, excepted for a few cell suspensions intoxicated with CCCP where up to 5% of the cells fell in the autofluorescence window, which is attributable to the total dissipation of the Δψ_m_, thus of oligomers formation, in these cells. The incorporation of CCCP led to the expected decrease of the oligomer/monomer (orange/green) fluorescences ratio (Fig. 4). Although 100 μM CCCP was used throughout the study, a dose-response study made in 2 horses suggested that maximal mitochondrial dysfunction is already reached with 1 μM.

**Figure 5:**
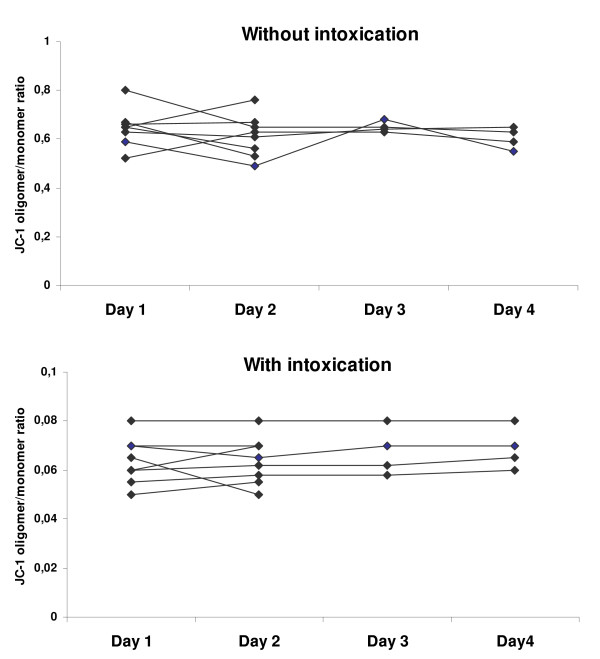
individual changes of JC-1 oligomer/monomer mean fluorescence ratio according to day of blood sampling. Eight horses were enrolled is this experimental setup (1, 7, 8, 10, 11, 15, 16, 23). JC-1 oligomer/monomer ratios were calculated from mean fluorescences generated by 10,000 PBMCs. There was no difference between days (P > 0.05).

**Figure 6:**
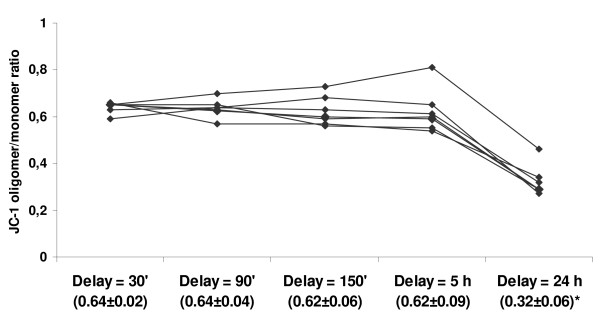
Effect of time elapsed between blood sampling and start of laboratory processing on equine PBMC JC-1 oligomer/monomer ratio. Seven horses were enrolled is this experimental setup (1, 7, 8, 10, 15, 16, 23). JC-1 oligomer/monomer ratios were calculated from mean fluorescences generated by 10,000 PBMCs (without intoxication). Delay refers to elapsed time between blood sampling and its processing into the laboratory. * Mean oligomer/monomer ratio was significantly different at 24 hours (P < 0.05).

**Figure 7:**
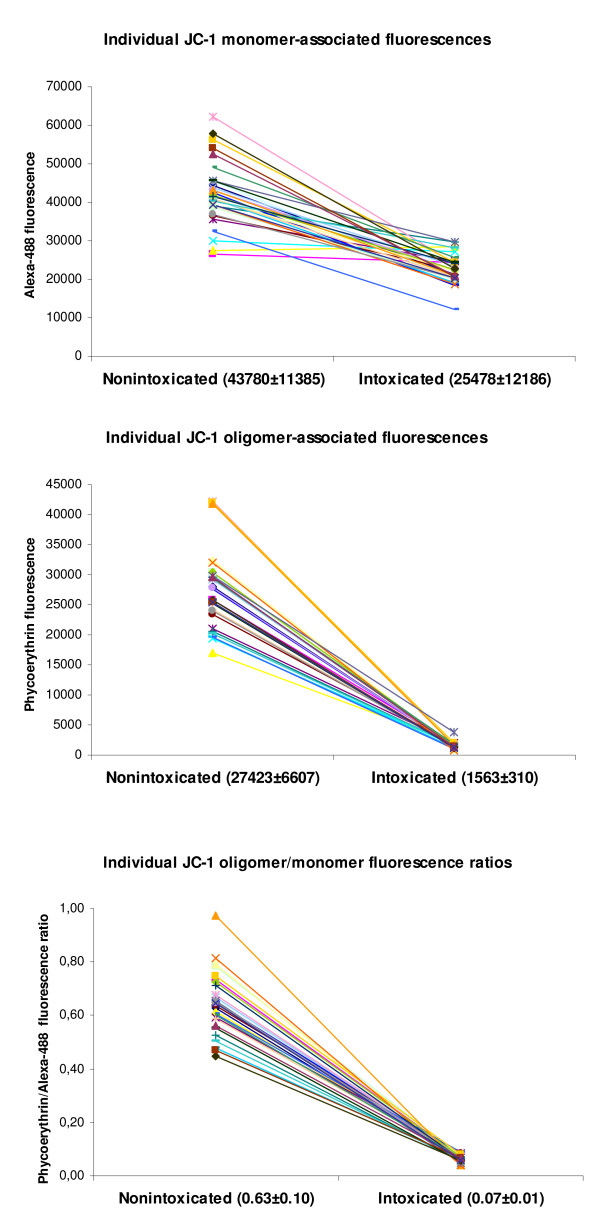
Individual JC-1 oligomer- and monomer-associated fluorescences and their ratio from PBMC retrieved from 31 clinically healthy horses.

Afterwards, mean absolute fluorescence values and ratio were measured in blood samples that had been kept at room temperature for different durations before laboratory processing (Fig. 5). No differences were seen up to 5 hours (P > 0.05). Also, when horses were sampled several times (1 to 35 days apart), the JC-1-associated fluorescence values retrieved from the corresponding PBMC populations (Table 1, Fig. 6) remained unchanged (P > 0.05). Regression of the orange/green ratio against age was not significant and sex-specific means of the ratio with (0.06 ± 0.01 in females vs. 0.06 ± 0.01 in neutered) and without intoxication (0.63 ± 0.13 vs. 0.67 ± 0.07) were not different (P > 0.05). Together, the 31 horses studied (Fig. 7) have generated mean values that can be taken as references : 42390 ± 8226 (monomer-associated fluorescence), 27385 ± 6391 (oligomer) and 0.64 ± 0.11 (oligomer/monomer ratio).

**Table 1:** Between-day reproducibility of JC-1 fluorescence in equine PBMC

	Without intoxication			With intoxication		
	Green* (monomer)	Orange* (oligomer)	Orange/green	Green* (monomer)	Orange* (oligomer)	Orange/Green

DAY 1 (n = 8)	43785 ± 2421	28461 ± 4895	0.65 ± 0.08	22159 ± 2643	1426 ± 108	0.06 ± 0.01
DAY 2 (n = 8)	45288 ± 3290	27766 ± 3796	0.61 ± 0.09	25144 ± 5357	1618 ± 389	0.06 ± 0.01
DAY 3 (n = 4)	47075 ± 6895	30789 ± 5418	0.65 ± 0.02	31141 ± 13145	2160 ± 1067	0.07 ± 0.01
DAY 4 (n = 4)	42055 ± 4729	25426 ± 2591	0.61 ± 0.04	25886 ± 13145	1812 ± 1067	0.07 ± 0.01

Horses contributing to days 1 and 2 were 1, 7, 8, 10, 13, 15, 16, and 23. Horses contributing to the four days were 1, 7, 8 and 16.* mean fluorescence values of 10,000 PBMCs are given in arbitrary units. Analysis of variance did not reveal any significant effect of the day of measurement (p > 0.1).

## Discussion

A method for the quantitative analysis of mitochondrial function in horse blood samples has been developed and validated. After PBMC purification and resuspension in PBS, cells are stained with JC-1 and analysed by flow cytometry. To our knowledge, this is the first report of mitochondrial function analysis with JC-1 in peripheral blood cells. We have shown that the homogeneity of cell staining and the signal-to-autofluorescence ratio are as favourable as they were in cell lines [12,16]. Interestingly, values measured after artificial intoxication are one order of magnitude (10 ×) lower than values measured from nontreated PBMC, which offers a large window to detect even subtle mitochondrial function changes. With respect to possible variation within each animal, we have shown that a clinically healthy horse generates similar mitochondrial function values over time. More, these values are independent of age and sex and were very homogeneous among the cohort of horses enrolled. Taken together, the present study therefore yields a reference value of mitochondrial function in equine PBMC, along with the calculation of the confidence interval in which 95% of healthy horses are predicted to fall: 0.64 ± 0.22 (oligomer-to-monomer ratio, mean ± 2SD). Should smaller animals, such as dogs and cats, be assayed, histopaque columns designed for smaller volumes must be used for purification and the final concentration of mononuclear cells suspensions should not be less than 200.000 per millilitre.

The starting point of this study consists in the compilation of several morphologic clues suggesting that a subset of equine myopathies might be attributable to mitochondrial dysfunction. These morphological suspicions have to be confirmed by functional data to gain credibility. Here, a new assay system is validated that, theoretically, could bring such functional confirmation. By "theoretically", it is meant that the assay will prove to be useful if and only if the mitochondrial dysfunction subjacent to a said clinical syndrome simultaneously affects circulating leucocytes. There is no relationship per se between myopathy and oxidative phosphorylation taking place in leucocytes, only the assumption that a mitochondrial myopathy is probably a polysystemic disease rather than a specific process affecting only the muscles. The polysystemic disease might clinically translate in a locomotor syndrome just because of the higher mitochondrial density in oxidative muscles.

## Conclusion

Whenever a quantitative measurement of mitochondrial function in a horse is desired, blood samples should be taken in sodium citrate tubes and kept at room temperature for a maximum of 5 hours before the laboratory procedure detailed here is started. This delay fits well with most field conditions, provided the samples must not be sent by post. We hope that the quantitative analysis of mitochondrial function procedure validated here will help in confirming, studying and preventing Equine Atypical Myopathy and toxic myopathies due to plant, bacterial and fungal mitochondrial toxins.

## Methods

### Animals

A group of 31 clinically normal male, female or neutered horses ranging between 8 months and 22 years of age, were enrolled into the study (Table 2). All animals were chosen at random. Eight of them were sampled on different days in order to evaluate the overall reproducibility of Δψ_m _measurements, i.e. the constancy of the orange/green fluorescence ratio when day, JC-1 working solution and laboratory handling are changed. In a second subset of horses (n = 7), five tubes of venous blood were collected at the same time. These tubes were kept at room temperature for variable durations (30 min., 90 min., 150 min., 5 h and 24 h) before laboratory processing was started, which permitted to assess whether and to what extent the delay between sampling and processing influences the value targeted.

**Table 2:** Breed, age and sex of the 31 horses enrolled in the study

ID	Breed	Sex	**Age (yr)**
1	**Warm blood**	**N**	15
2	**Warm blood**	**N**	16
3	**Warm blood**	**N**	10
4	**Warm blood**	**F**	5
5	**Warm blood**	**F**	3
6	**Warm blood**	**N**	19
7	**Shire**	**F**	13
8	**Warm blood**	**F**	14
9	**Warm blood**	**F**	14
10	**Warm blood**	**F**	6
11	**Warm blood**	**N**	5
12	**Warm blood**	**F**	7
13	**Standard bred**	**F**	14
14	**Spanish horse**	**N**	12
15	**Warm blood**	**N**	5
16	**Frisian**	**F**	7
17	**Warm blood**	**N**	8
18	**Warm blood**	**F**	22
19	**Warm blood**	**N**	11
20	**Warm blood**	**F**	10
21	**Warm blood**	**F**	5
22	**French saddle horse**	**F**	17
23	**Warm blood**	**F**	10
24	**Warm blood**	**M**	7
25	**Fjord**	**F**	14
26	**Standard bred**	**F**	1
27	**Standard bred**	**F**	1
28	**Warm blood**	**F**	1
29	**Cross-bred Arab horse**	**F**	1
30	**Warm blood**	**F**	2
**31**	Warm blood	F	2

N, neutered; M, male; F, female

### Chemicals

JC-1 (5,5',6,6'-tetrachloro-1,1',3,3'-tetraethylbenzimidazolylcarbocyanine iodide) was from Molecular Probes (Eugene, OR, USA) and carbonyl cyanide m-chlorophenylhydrazone (CCCP) was purchased from Sigma-Aldrich (Sigma Immunochemicals, St. Louis, MO, USA). Working solutions, 200 μM (JC-1) and 50 μM (CCCP), both in DMSO, were prepared and equilibrated to room temperature in advance.

### Isolation of mononuclear cells

Five milliliters of venous blood was collected in BD Vacutainer sodium citrate 0.105 M and kept at room temperature until processing. PBMC were separated from the blood by layering it over histopaque (density: 1.077 ± 0.001) and centrifuging (1200 g for 20 min). The cell content and the purity of the preparations were checked by visual inspection using light microscopy. The viability of the cells was controlled by flow cytometry with propidium iodide staining and was found to exceed 95%. For flow cytometric PBMC autofluorescence and JC-1-associated fluorescence determinations, the mononuclear cells were then suspended in azid-free PBS at a final concentration of ~10^6 ^cells per millilitre.

### Visualization of changes in the Δψ_m_

For each horse, one PBMC-containing 1 ml aliquot was used for autofluorescence measurements, and two others for Δψ_m _determinations with or without intoxication with the Δψ_m _dissipator CCCP. JC-1 staining was as follows: 1 ml of the PBMC suspension in PBS was incubated at 37°C for 10 min, stained with 10 μl of JC-1 working solution (2 μM final concentration) followed by another 25 min at 37°C under a carbon dioxide-enriched atmosphere (5% in air). Cells were then washed and the pellet was resuspended in PBS for flow cytometric analysis. For intoxication, 2 μl of the CCCP working solution was incorporated into the PBMC suspension during the first incubation period (100 μM final concentration).

### Flow cytometry

Autofluorescence and JC-1-dependent fluorescence changes were recorded using a FACSCanto flow cytometer (Becton-Dickinson) using 488 nm excitation with 530/30 nm (FL1, green) and 585/42 nm (FL2, orange) bandpass emission filters. The reproducibility of the fluorescence readings of the instrument was proven before all measurements using fluorescein isothiocyanate- and R-phycoerythrin-labeled polymethylmethacrylate microspheres (BD FACS 7-color Setup beads, Becton-Dickinson). The sample flow rate was adjusted to about 1000 cells/s. For one single analysis, the fluorescence properties of 10,000 mononuclear cells were collected. The respective gates were defined using the distinctive forward-scatter and side-scatter properties of the individual cell populations. The data were analyzed using the FacsDIVA software (Becton-Dickinson). After JC-1 staining, there were always less than 1 and 5% of cells within the green and orange predefined autofluorescence intervals respectively.

### Statistical analysis

The results are presented as means ± SD. Significant changes were assessed by Student's *t *test. A value of P < 0.05 was accepted as the level of significance.

## List of abbreviations

JC-1, 5,5',6,6'-tetrachloro-1,1',3,3'-tetraethylbenzimidazolylcarbocyanine iodide; Δψ_m_, mitochondrial membrane potential; CCCP, carbonyl cyanide m-chlorophenylhydrazone; PBMC, peripheral blood mononuclear cells; FSC, forward scatter; SSC, side scatter.

## Competing interests

The author(s) declares that there are no competing interests.

## Authors' contributions

DC carried out the blood samplings, transportation and PBMC isolation. TF carried out flow cytometry calibration and analyses. MS carried out cell culture, intoxication and staining. DC, EB and FC participated in the design of the study and helped to structure analysis. DD conceived the study, participated in its design and coordination and drafted the manuscript.
